# Normalized vitamin D metabolite concentrations are better correlated to pharmacological effects than measured concentrations

**DOI:** 10.4155/fso.15.83

**Published:** 2015-11-30

**Authors:** Darius Mason, Paul J Donabella, Daryl Nnani, Florin Marcel Musteata

**Affiliations:** 1 Department of Pharmacy Practice, Albany College of Pharmacy & Health Sciences, 106 New Scotland Avenue, Albany, NY 12208, USA; 2 Division of Nephrology & Hypertension, Albany Medical College, 25 Hackett Boulevard, Albany, NY 12208, USA; 3 Department of Pharmaceutical Sciences, Albany College of Pharmacy & Health Sciences, 106 New Scotland Avenue, Albany, NY 12208, USA

**Keywords:** 1,25-dihydroxyvitamin D, 25-hydroxyvitamin D, normalized concentrations, vitamin D binding protein, vitamin D metabolites

## Abstract

**Background:**

Vitamin D deficiency has been associated with a multitude of diseases, ranging from fractures to cancer. Nearly 99% of vitamin D metabolites are bound to proteins, altering the relationship between concentration and activity.

**Methods & results:**

Normalized concentrations were calculated and validated using published data regarding the correlation of 25-hydroxyvitamin D with bone mineral density. In addition, healthy and kidney disease subjects were recruited for preliminary investigations. Use of the normalizing equations resulted in statistically significant improvements in the relationship between vitamin D metabolites and several markers of health status.

**Conclusion:**

Normalized concentrations are similar to clinically reported values and are easier to interpret than free or bioavailable concentrations, since their values match the range of measured total concentrations.

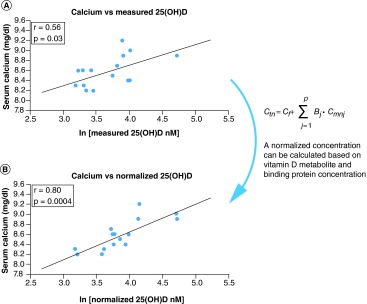

## Abstract (lay abstract)

**Lay abstract:** Vitamin D deficiency has been associated with a multitude of diseases, ranging from fractures to cancer. Most vitamin D metabolites are bound to various body components, altering the relationship between reported concentration and biological effects. To compensate for differences in binding between individuals, normalized concentrations were calculated. Use of the normalizing equations resulted in significant improvements in the relationship between the concentration of vitamin D metabolites and health status. The newly developed normalized concentrations are therefore better indicators of vitamin D status and are easier to interpret than free or bioavailable concentrations, since their values match the range of measured total concentrations.

**Figure 1.**
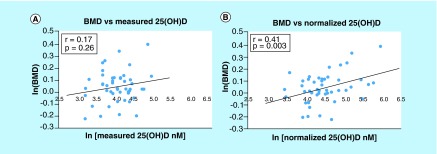
**Comparison of the correlations between bone mineral density.** **(A)** Measured 25(OH)D, and **(B)** normalized 25(OH)D; each point represents one set of measurements from one volunteer; measured vitamin D was obtained by direct analysis of the blood sample; normalized vitamin D was obtained from measured vitamin D and the concentration of binding proteins; the line represents the best linear fit. BMD: Bone mineral density. Adapted with permission from [13].

**Figure 2.**
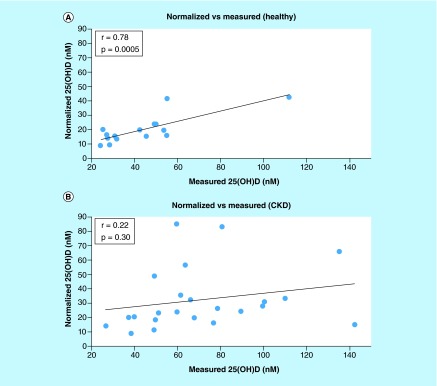
**Measured concentration of total 25(OH)D and the resulting normalized concentration of 25(OH)D calculated with Equation 2.** **(A)** Healthy and **(B)** CKD volunteers; each point represents one set of measurements from one volunteer; measured vitamin D was obtained by direct analysis of the blood sample; normalized vitamin D was obtained from measured vitamin D and the concentration of binding proteins; the line represents the best linear fit. CKD: Chronic kidney disease.

**Figure 3.**
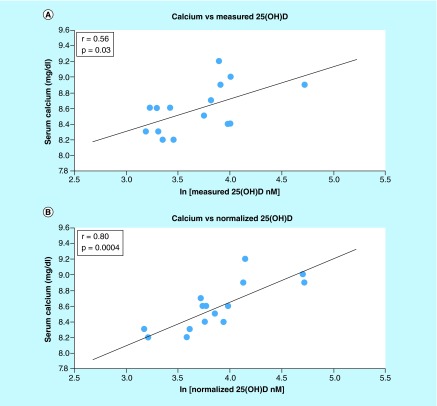
**Correlation of serum calcium with ln(25(OH)D) for the healthy subjects.** **(A)** Measured and **(B)** normalized metabolite concentration; each point represents one set of measurements from one volunteer; measured vitamin D was obtained by direct analysis of the blood sample; normalized vitamin D was obtained from measured vitamin D and the concentration of binding proteins; the line represents the best linear fit.

Vitamin D is a family of compounds that contains more than 40 different metabolites [1]. Almost all mammalian cells contain the vitamin D receptor and a large fraction of the human genome is regulated by a vitamin D related pathway, making this vitamin one of the most essential in the human body [2]. In this article, the general term ‘vitamin D' refers to cholecalciferol as well as the hydroxylated metabolites unless specifically stated. For most human beings, vitamin D intake is obtained partly from sun exposure and partly from diet [3–5]. In the northern hemisphere, vitamin D deficiency has become a common diagnosis over the past years mostly due to reduced sunlight exposure. The patients most at risk for vitamin D deficiency are the elderly, infants, residents of northern latitudes, patients with darker skin pigmentation, renal or liver disease patients and obese patients [6]. The main function of vitamin D is to serve as a component of the biological apparatus whereby cells access the information in the genome to carry out cell-specific functions [7]. Recent evidence has shown that the active metabolites of vitamin D may be able to regulate different cellular processes associated with carcinogenesis [8]. Maintaining an optimal level of vitamin D may help in preventing the risk of cancer, mental disease, depression and asthma [1,9–12].

In order to exert biological activity, the vitamin needs to be converted into 25-hydroxyvitamin D [25(OH)D] and then into the active metabolite 1,25-dihydroxyvitamin D [1,25(OH)_2_D]. More than 99% of vitamin D and its metabolites circulating in the bloodstream are bound to plasma proteins. The binding of vitamin D metabolites to various plasma proteins changes the relationship between concentration and biological effects [9,13]. Different people have widely different concentrations of plasma binding proteins [14], and therefore the same concentration of vitamin D can produce very different health outcomes. The free hormone hypothesis states that only hormones free from binding protein are capable of entering the cell and exerting biological activity [13]. While 25(OH)D can enter some cells through the megalin-cubilin mechanism, most biological effects are still found to be more closely related to its free concentration [13,15–16].

Many disease states, as well as advanced age, result in reduced liver synthesis which leads to low levels of albumin and other plasma proteins [17,18]. Depending on the concentration of plasma proteins and their genetic polymorphism, the free concentration of drugs and hormones can change unpredictably [17,19–23]. This interindividual variability in free concentrations and protein binding makes interpretation of total concentrations complicated, contributes to the difficulty in determining vitamin D status, and is most likely the cause for several conflicting reports of vitamin D associations [24]. The plasma proteins that vitamin D compounds bind to most commonly are albumin and vitamin D binding protein (DBP).

Vitamin D binding protein, also known as group-specific component (Gc-globulin), is a single chain polypeptide with a molecular weight of about 58 kDa [25–27]. Levels of DBP remain stable from birth with limited deviation; any major changes in DBP concentrations can be attributed to hepatic diseases or pregnancy, as there is no evidence of seasonal variations [25,27–28]. DBP is the only protein that is capable of binding to vitamin D, vitamin D metabolites and G-actin with a high affinity [25,29]. It has been observed that 85–90% of circulating vitamin D metabolites are bound to DBP and 12–15% are bound to albumin [25,30–31]. The higher percentage bound to DBP is due to a much higher affinity towards the metabolites. The most commonly used affinities of DBP and albumin towards cholecalciferol metabolites can be found in Table 1.

Vitamin D status in the body is commonly determined by measuring the total concentration of 25(OH)D [10,34–36]. This metabolite is found in the blood at nearly 1000 times greater concentration than the biologically active metabolite 1,25(OH)_2_D and is relatively more stable [5]. The half-life of 25(OH)D is about 3 weeks, making it much easier to measure than 1,25(OH)_2_D, which has a half-life of only about 4 h [37]. Analysis of 1,25(OH)_2_D is commonly limited to patients that suffer from diseases where vitamin D status needs to be more closely monitored such as lymphoma, hypercalcemia, sarcoidosis or other granulomatous diseases, and rickets [10].

The optimal level of 25(OH)D is uncertain and varies depending on the different stages of life and whether skeletal or nonskeletal outcomes are examined [6]. Current guidelines state that 25(OH)D levels around 75 nM are sufficient to maintain a healthy status, whereas levels less than 37 nM are insufficient. However, there are many different opinions on what the ‘optimal’ or sufficient concentration of 25(OH)D should be, with some practitioners recommending levels as high as 150 nM [38]. Health risks caused by low concentrations of vitamin D have become a popular topic in the scientific community and there is a growing public awareness of these health risks which, in turn, has created strain on laboratories and a need for analytical method improvement due to increased scrutiny and controversy in an effort to interpret the results of analysis. The current difficulty in establishing optimal vitamin D levels is partly caused by weak correlations with clinical outcomes or other biomarkers [1,9,13]. Recently, it has been shown that some of these low correlations are caused by interpatient variability in albumin and DBP. Furthermore, current assays for measurement of 25(OH)D and 1,25(OH)_2_D measure both bound and unbound metabolites and results may be affected by changes in DBP concentrations [39]. Some newer publications are proposing that vitamin D status should be based on measurement of free 25(OH)D [40]. Recent modeling efforts and clinical studies strongly support the need to account for changes in free concentrations and plasma protein binding between patients [14], but there is no quantitative basis for these adjustments.

Measuring the concentration of bioactive compounds is essential for optimizing pharmacotherapy and provides the basis for studies involving pharmacokinetics and pharmacodynamics. To compensate for differences between patients, a normalized concentration of the bioactive compound can be calculated. The normalized vitamin D metabolite concentration for a particular patient would be the concentration that produces a similar pharmacodynamic effect in an individual with average body composition in which the normal levels of various markers are usually established. One of the most widely accepted ways to normalize concentrations for a particular individual is based on calculating the total concentration that would generate the same free concentration in an individual with average plasma protein binding. From our previous work [14] it has been determined that the normalized concentration *C_tn_* can be calculated as:
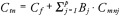



where *C_f_* is the free vitamin D metabolite concentration, *p* is the number of binding proteins, *j* is one of the proteins, *B_j_* is the number of molecules of vitamin D metabolite bound per molecule of the *j^th^* protein and *C_mn j_* is the normal (average) concentration of protein *j*.

The objective of this study is to develop the first equations for normalizing the concentrations of 25(OH)D and 1,25(OH)_2_D. The normalized values can be obtained based on either calculated or measured free concentrations (Equation 1). The normalizing equations are expected to compensate for interindividual variability in plasma protein binding and to provide concentration values that are easier to interpret than free or bioavailable concentrations. The equations could have the potential for standardization of population pharmacokinetics and pharmacokinetic-pharmacodynamic modeling for vitamin D and its metabolites.

## Materials & methods

### Normalizing equation

Vitamin D metabolites bind to two proteins, each with one binding site. Using the model ‘Drug that binds to two proteins’ described in our previous work [14], the equation for determining the normalized concentration was set up as
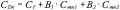



where *C_Dn_* is the normalized vitamin D metabolite concentration, *C_f_* is the free vitamin D metabolite concentration, *B_1_* is the number of molecules of vitamin D metabolite bound per molecule of albumin, *B_2_* is the number of molecules of vitamin D metabolite bound per molecule of DBP, *C_mn 1_* is the normal (average) concentration of albumin (considered to be 662 µM in this article), and *C_mn 2_* is the normal concentration of DBP (5.7 µM in this article). The values for average protein concentrations were obtained from previous larger studies [14,16,41].

The values of *C_f_*, *B_1_*, and *B_2_* can be determined from the following equations:
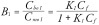


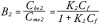


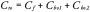



where *K_1_* is the binding constant of the vitamin to albumin, *K_2_* the binding constant to DBP, *C_bo1_* is the concentration of vitamin bound to albumin, *C_bo2_* is the concentration of vitamin bound to DBP, *C_mo1_* is the concentration of albumin in the sample and *C_mo2_* is the concentration of DBP in the sample.

The values of the binding constants used for the normalizing equations can be found in Table 1; this information was obtained from previously published research [32,33].

Using Equations 3, 4 & 5, a system can be formed with three unknowns that can be solved for the free drug concentration (*C_f_*) in the sample. Alternatively, the free concentration value can be measured in the sample. Subsequently, the values of *B_1_* and *B_2_* can be calculated and plugged into Equation 2 to compute the normalized concentration of vitamin D metabolite in any sample. More detailed equations are given in the Supplementary Data file.

### Relationship between the concentration of 25(OH)D & bone mineral density

The normalizing equation was first applied to data from a previously published paper, where the authors examined the relationship between vitamin D, DBP, and bone mineral density (BMD) [13]. The authors provided values for free 25(OH)D concentrations which were used to calculate B_1_ and B_2_ with Equations 3 & 4. Subsequently, the normalized 25(OH)D concentration was calculated with Equation 2 and plotted versus BMD. Statistical analyses were performed using Microsoft Excel 2013. All statistical tests were performed at a two-sided 0.05 level of significance.

### Blood collection & sample analysis

Blood samples from healthy volunteers (n = 15) were collected and analyzed for calcium, phosphate, albumin, alkaline phosphatases, parathyroid hormone (PTH), total cholesterol, total 25(OH)D and DBP. The blood samples taken from the healthy volunteers were collected near the end of winter in the northeast USA. All of the healthy volunteers were college students with normal physical activity levels. None of the healthy volunteers took vitamin D supplementation. BMI over 25 was considered overweight. After an overnight fast, blood samples of approximately 15 ml were obtained in a vacuum blood collection tube from each subject by a licensed phlebotomist. Both serum and plasma portions were collected, made into aliquots, and stored at -80°C. Blood samples of chronic kidney disease (CKD) patients (n = 23) were collected from a previous study, with similar blood collection parameters. None of the CKD patients received any ergocalciferol or cholecalciferol amounts greater than 2000 units for at least 3 month as a requirement for clinical study inclusion. Clinical parameters were measured by standard techniques. Total 25(OH)D measurements were performed by the Diasorin Liaison direct competitive chemiluminescence immunoassay for the CKD subjects and Siemens Centaur XP for the healthy subjects (the two sets of samples were obtained at different times and different funding sources were available for analysis). The concentration of 1,25(OH)_2_D, available for CKD subjects only, was measured by LC-MS/MS at LabCorp, Albany, NY. Vitamin D binding protein levels was determined by ELISA, R&D Systems (MN, USA; Catalog Number DVDBP0) per assay protocol. Creatinine clearance was determined by the Cockcroft–Gault equation. This study was approved by the Institutional Review Boards at Albany College of Pharmacy and Health Sciences. The study was conducted in accordance with the ethical principles that have their origin in the Declaration of Helsinki. Written informed consent was obtained from all participants prior to conducting any study procedures.

### Statistical analyses

Data for all continuous variables were summarized and reported as mean ± standard deviation or as median and interquartile range for skewed variables. Between groups (healthy versus CKD) comparisons of demographics were made using the independent-sample t-test or Wilcoxon rank-sum test as appropriate. Pearson's χ^2^ test and Fisher's exact test were used to analyze categorical variables where appropriate. 25(OH)D and 1,25(OH)_2_D levels were log transformed before statistical analysis. Pearson's correlation coefficients were calculated to investigate associations between vitamin D metabolites (measured and normalized) and clinical continuous variables. Statistical analyses were performed using SAS version 9.3 (SAS Institute Inc., NC, USA).

## Results

### Relationship between the concentration of 25(OH)D & bone mineral density

Powe *et al.* investigated the relationship between total and free 25(OH)D and lumbar spine bone mineral density (BMD). The authors found the free concentration as well as the bioavailable 25(OH)D (free plus albumin-bound compound) demonstrate better correlation to BMD than measured 25(OH)D levels [13]. By applying the normalization equation to their data, a similar improvement in correlation and significance was found, as shown in Figure 1. Normalized concentrations corresponding to free concentrations can be calculated using both albumin and DBP as binding proteins. Normalized concentrations corresponding to bioavailable concentrations can be calculated using only DBP as the binding protein.

### Subjects for preliminary study

Fifteen healthy subjects and 23 CKD patients were included in the analysis with an average age of 24.5 ± 2.6 and 67.4 ± 7.3, respectively (Table 2). CKD subjects had significantly higher calcium, creatinine, parathyroid, glucose and blood urea nitrogen concentrations than healthy subjects. The estimated creatinine clearance was significantly higher in the healthy population. The average measured 25(OH)D for the healthy volunteers, 17.5 ng/ml, was lower than the CKD group. While the vitamin D concentrations were lower in the healthy group of volunteers, it is not surprising to find low vitamin D levels in young healthy individuals residing in northern states [42], especially near the end of winter. DBP concentrations ranged from 1.2 µM to 22.1 µM. Although there are some concerns regarding the accuracy of the method for measuring the levels of DBP in blacks, our study population included only one black in the CKD group. As better assays for measuring DBP become available, it is likely that the correlations reported in this paper will improve. Also, given the low number of participants, no provisions were made for differing genotypes – especially considering that some studies indicate similar affinity of DBP genetic variants for vitamin D metabolites [43]. Furthermore, the sensitivity of the resulting normalized concentrations to changes in the binding constants is rather small; the results are similar even with binding constants that are 2–3 times different from those in Table 1. PTH levels in the CKD volunteers were significantly higher than in healthy subjects.

#### Measured vs normalized 25(OH)D levels

The calculated normalized concentrations and the observed concentrations for 25(OH)D pertaining to each group are shown in Figure 2. The normalized concentrations demonstrated a significant correlation with the measured values in the healthy subjects. However, for the CKD subjects, measured 25(OH)D versus normalized 25(OH)D plotted values were fairly scattered and did not reveal a significant relationship.

### Correlation between normalized vitamin D metabolites & clinical measures

The relationships between common clinical measurements and the concentration of 25(OH)D (measured and normalized) were determined in the healthy subjects. Normalized 25(OH)D values significantly improved correlations for serum calcium (from r = 0.56, p = 0.029 to r = 0.80, p = 0.00037), parathyroid hormone (from r = 0.46, p = 0.084 to r = 0.56, p = 0.028) and BMI in overweight individuals (from r = 0.76, p = 0.24 to r = 0.997, p = 0.0029), as shown in Table 3. Although calcium demonstrated a significant correlation with measured 25(OH)D, normalization further strengthened the relationship demonstrating a significantly improved linearity (Table 3 & Figure 3).

Correlations between 25(OH)D and clinical measurements in CKD subjects achieved statistical significance for calcium and PTH plus calcium only after adjustment for creatinine clearance (Table 4). Similar results and improvements in clinical parameters relationships after adjustment for creatinine clearance were also seen for normalized 1,25(OH)_2_D (Table 5).

## Discussion

### Validation

To validate the newly proposed normalization equations, they were initially applied to data found in other publications where the authors provided sufficient information to allow for calculating the normalized concentration of vitamin D metabolites. Based on the data shown in Figure 1, the newly proposed metric of vitamin D status (normalized concentration) was as good at improving correlations as previously published methods based on free or bioavailable concentrations. This was not unexpected, since this initial attempt at normalizing the concentrations of vitamin D metabolites was based on equalizing the free concentrations between the investigated patient and the ‘average patient.’ Therefore, all previous publications reporting improved correlations for free or bioavailable vitamin D metabolite concentrations [9,13,15–16,19,40] are in support of the newly proposed method for normalizing the vitamin D concentration.

Direct measurement of free concentrations is expensive, labor intensive, time consuming, and suffers from low accuracy – especially when based on immunoassays. While other investigator equations return a bioavailable concentration, that is, orders of magnitude different from the total concentration [13,24], the newly proposed normalizing equation returns values in the same range as the measured total concentration. All previous equations for calculating free or bioavailable vitamin D metabolites are based on simplistic models for evaluating the free fraction of testosterone in serum [40]. Some other advantages of the new normalizing equation over previous methods include straightforward inclusion of any binding model and any number of binding proteins as well as considerable flexibility in adding new parameters to the procedure. Furthermore, the normalized concentrations can also be obtained based on measured free concentrations when they are available [40], which might further improve the observed association with pharmacological effects.

Description of the interaction between vitamin D metabolites and specific human plasma proteins was based on a thorough review of the data available in current scientific literature. The concentration of plasma proteins is dependent on factors such as age, genotype, nutritional status and disease state. The normalized concentration of a compound for a particular patient is the concentration that would produce a similar pharmacodynamic effect in an individual with average body composition. This is important as there is a shift towards more personalized treatment options for patients with health problems. The normalizing equation calculates the corrected concentration of vitamin D metabolite based on three main factors: average protein levels expected in a healthy individual in which normal marker values are established, compound concentration (free or total, as available) and the number of molecules of drug bound per molecule of protein at equilibrium in the plasma of a specific patient. The concentration of DBP has a higher influence in the equation than albumin, since vitamin D metabolites have a higher affinity for DBP than they do for albumin. The concentration range of DBP is much wider than the concentration range of albumin, further increasing the influence of DBP on the normalized concentration. Nevertheless, the concentration of albumin in plasma is about 100 times higher than that of DBP, making it necessary to include it in the calculation of the normalized concentration.

The normalization procedure is applied the same way regardless of the vitamin D metabolite and protein concentrations; however, the correlations with effects might be better at certain concentrations and certain effect levels, since the relationship between logarithm of concentration and effect is linear only between 20 and 80% of maximum effect.

### Preliminary study

In our preliminary studies on a small group of volunteers, of all the markers investigated, calcium saw the greatest increase in correlation in the healthy individuals when the normalized concentration was used. Based on r^2^ values (0.31 for measured and 0.64 for normalized 25(OH)D), the normalized concentration was at least two times better than the measured concentration at explaining the variability in serum calcium among volunteers. Positive linear associations between ln[25(OH)D] and calcium have also been previously reported by several other studies based on much larger sample sizes [13,19,40,44]. We hypothesize that such correlations were not observed more often since most studies so far overlooked the influence of albumin and DBP on the association. The interassay variability caused by using different platforms for analysis of vitamin D metabolites for the healthy and CKD volunteers was not an issue since the two sets of data were analyzed independently of each other.

Accumulation of uremic toxins [45] and changes in acid-base status [46] in CKD interfere with homeostatic protein binding. These pathologic changes result in poor or nonexistent correlations for commonly associated protein bound markers such as calcium in CKD. Such disassociated relationships could be attributed to our lack of association between calcium and measured 25(OH)D seen in our study population of CKD patients. To account for the degree of impaired binding abilities, CKD patients’ markers were adjusted for kidney function. Significant improvements in association between normalized 25(OH)D and 1,25(OH)_2_D with calcium with kidney adjustment illustrates the flexibility of our equations to adjust for common disease states that might influence protein binding. A lack of association with PTH alone for the measured or normalized vitamin D values is not unexpected [47]. In CKD, secondary pathologies further complicate the vitamin D-PTH-Calcium axis. Thus, combining the PTH and calcium together and adjusting for kidney function provided a stronger association with normalized 25(OH)D values. DBP has the greatest affinity for 25(OH)D, whereas due to steric hindrance of the additional hydroxyl group, the affinity for 1,25(OH)_2_D is 10- to 100- folds lower [43]. Yet, similar results were seen with marker values adjusted for kidney function and normalized 1,25(OH)_2_D, albeit less significant associations.

Lipid soluble properties of vitamin D metabolites allow for their sequestration within excess adipose tissue. Obesity is commonly considered a risk factor for vitamin D deficiency in health and disease. The influence of increased volume of vitamin D distribution seen in obesity can obscure the relationship between vitamin D concentrations and biological activity when excessive weight has not been standardized. Furthermore, obesity may play a role in reducing vitamin D bioavailability and serum concentrations [48]. Our normalized 25(OH)D values significantly enhanced the correlations with BMI in overweight individuals.

## Conclusion

The problems associated with measuring 25(OH)D and the growing interest in determining the optimal levels and dosing regimens for vitamin D led to this research. The main objective was to develop better correlations between markers of health status and vitamin D concentrations. While several equations have been published for calculating the free concentration and the bioavailable concentration of 25(OH)D [13,40,45], they are based on simplistic binding models for *testosterone* and return concentrations that are significantly outside the range of the measured 25(OH)D. In turn, this makes data interpretation and comparison of different results from various studies difficult. The current approach for normalizing the concentration of 25(OH)D includes the influence of plasmatic proteins on the distribution of the compound in the body and is, therefore, a more accurate interpretation than the measured concentration, making it easier to compare values and pharmacological effects between patients.

Using data from previously published research articles showed that the normalized concentration of 25(OH)D is as useful as the free or bioavailable fraction in interpreting vitamin D status. The use of this equation and the correlations that were created can lead to a more meaningful exploration of the vitamin D status in various populations. This, in turn, will allow for a more personalized treatment or supplementation plan for patients based on their individual concentration of plasma proteins, possibly resulting in decreased toxicity associated with vitamin D and better health outcomes. Our results suggest that normalized vitamin D concentrations offer better relationships with clinical status than the measured total vitamin D concentrations. Considerations should be given to patients’ normalized values within the established reference range. It should also be noted that different pharmacological effects may have different vitamin D requirements; accordingly, a different reference range of vitamin D metabolite concentrations should be established for each effect.

The main advantages of our approach are: easy comparison with other publications based on total concentrations; returns values that are in the same range as the results of total concentration measurement done in the clinical lab – facilitating integration with previous guidelines regarding optimal levels of vitamin D metabolites; better for comparing the values in different patients (because of normalization); better for calculating dosage regimens (based on volume of distribution for total concentration); and can be easily adjusted to include other factors that influence the biological activity of vitamin D (such as the megalin-cubilin mechanism). The newly proposed method for normalizing the concentration of vitamin D metabolites is applicable for any concentration values for metabolites and binding proteins [14].

Some limitations in our investigation are worthy of mention. Genetic polymorphisms in the DBP result in altered protein binding [49] and highly variable DBP levels [24]. Although genetic variants were not determined in this study, the new equations do account for the DBP levels which may alleviate partially the need for genotyping to determine likelihood of having low or high DBP levels. Newer more accurate measurement of the binding constants for vitamin D metabolites with various DBP variants and albumin could strengthen this research and most likely improve the correlations. Interestingly, the literature published within the past 5 years, including the current study, still uses binding constants determined by Bikle *et al.* in 1986.

## Future perspective

The results presented here can be a useful guide for future research done with a larger number of subjects which will increase the statistical power. Furthermore, investigators with available vitamin D, albumin and DBP data can easily incorporate their values into the normalization equations for comparison. Incorporation of protein binding characteristics in interpretation of laboratory measurements has value for improving vitamin D research, minimizing interpatient variability, and improving vitamin D assessments. If the improved correlations between the normalized concentrations of vitamin D metabolites and clinical effects are confirmed in large clinical trials, the proposed normalization procedure will likely become the standard approach for interpreting the vitamin D status.

**Table 1.** The binding constants of vitamin D_3_ metabolites to vitamin D binding protein and albumin [32,33].

**Binding constant (K_a_)**	**DBP**	**Albumin**
25(OH)D_3_	7.0 × 10^8^ M^-1^	6.0 × 10^5^ M^-1^
1,25(OH)_2_D_3_	3.7 × 10^7^ M^-1^	5.4 × 10^4^ M^-1^

**Table 2.** Demographic information for the study groups.

**Parameter/mean ± SD, n (%)**	**Healthy (n = 15)**	**CKD (n = 23)**	**p-value**
Age (years)	24.3 ± 2.6	67.4 ± 7.3	<0.0001
BMI	26 ± 7	36 ± 7.7	0.0003
Weight (lbs)	166.9 ± 55.2	196.8 ± 75.4	0.2
Gender:			0.03
– Male	7 (47%)	19 (83%)	
– Female	8 (53%)	4 (17%)	
Race:			0.1
– White	13 (87%)	22 (96%)	
– Nonwhite	2 (13%)	1 (4%)	
Albumin (g/dl)	3.97 ± 0.36	3.85 ± 0.23	0.2
Calcium (mg/dl)	8.6 ± 0.30	9.4 ± 0.53	<0.0001
Blood urea nitrogen	11.2 ± 2.8	29.3 ± 9.9	<0.0001
Cholesterol	169 ± 32	161 ± 34	0.5
25(OH)D (nM)	43.8 ± 22	70.9 ± 30	0.005
DBP (µM) median [interquartile]	4.9 ± 1.5 5.0 (4.0–6.0)	6.29 ± 4.4 5.4 (3.9–7.9)	0.18 0.50
PTH (pg/ml	41 ± 16	65 ± 40	0.01
Creatinine (mg/dl)	0.7 ± 0.09	1.8 ± 0.49	<0.0001
Creatinine clearance (ml/min/1.73m^2^)	121 ± 28	40 ± 14	<0.0001
Glucose (mg/dl)	82 ± 16	143 ± 54	<0.0001

CKD: Chronic kidney disease; DBP: Vitamin D binding protein; PTH: Parathyroid hormone; SD: Standard deviation.

**Table 3.** Correlations between 25(OH)D and characteristics of the healthy subjects.

**Parameter**	**Measured 25(OH)D**		**Normalized 25(OH)D**	
	**r**	**p-value**	**r**	**p-value**
Calcium	0.56	0.029^†^	0.80	0.00037^†^
PTH	0.46	0.084	0.56	0.028^†^
BMI (normal)	0.029	0.93	0.44	0.17
BMI (overweight)	0.76	0.24	0.997	0.0029^†^

Levels of 25(OH)D were log transformed before analysis.

^†^Indicates statistical significance.

PTH: Parathyroid hormone.

**Table 4.** Correlations between 25(OH)D and characteristics of the chronic kidney disease study population.

**Parameter**	**Measured 25(OH)D**		**Normalized 25(OH)D**	
	**r**	**p-value**	**r**	**p-value**
Calcium	0.14	0.51	0.2	0.34
Calcium adjusted for Cl_Cr_	0.14	0.52	0.49	0.017^†^
PTH	0.1	0.65	0.18	0.42
PTH and calcium	0.27	0.46	0.36	0.25
PTH and calcium adjusted for Cl_Cr_	0.27	0.47	0.63	0.007^†^

Levels of 25(OH)D were log transformed before analysis.

^†^Indicates statistical significance.

**Table 5.** Correlations between 1,25(OH)_2_D and characteristics of the chronic kidney disease study population.

**Parameter**	**Measured 1,25(OH)_2_D**		**Normalized 1,25(OH)_2_D**	
	**r**	**p-value**	**r**	**p-value**
Calcium	0.047	0.83	0.3	0.17
Calcium adjusted for Cl_Cr_	0.053	0.81	0.48	0.02^†^
PTH	0.056	0.80	0.0016	0.99
PTH and calcium	0.065	0.96	0.31	0.37
PTH and calcium adjusted for Cl_Cr_	0.091	0.92	0.51	0.05^†^

Levels of 1,25(OH)_2_D were log transformed before analysis.

^†^Indicates statistical significance.

**Executive summary**

The normalized concentration corresponds to drug effect in an ‘average’ individual.
Normalizing equations improve the relationship between vitamin D and health status.
The greatest improvements in correlation were found for 25(OH)D versus calcium.
Normalized concentrations are easier to interpret clinically than free values.
This approach can offer more personalized interpretation of vitamin D levels.
