# Supplementary material for: Normalized vitamin D metabolite concentrations are better correlated to pharmacological effects than measured concentrations
Source: Future Sci OA. 2015 Nov 30;1(4):FSO83. doi: 10.4155/fso.15.83 (PMC5137860; doi:10.4155/fso.15.83)
Supplement: Supplementary file 1 [file fso-01-83-s1.docx]

*Normalizing equation calculation and application*

In order to create a normalizing equation all data had to be collected from all of the patients. Binding constants were determined from scientific literature. The binding constants used for the normalizing equation can be found in table 1. There is not much current research providing the needed binding constants. Literature published within the past 5 years still uses these numbers for the binding constants. Using the model “Drug that binds to two proteins,” explained in reference 14 from the main article, the equation for determining the normalized concentration was set up as;

$C_{Dn}= C_{f}+ B_{1}* C_{mn_{1}}+ B_{2}* C_{mn_{2}}$ (Equation 1)

Equation 1 is beneficial when a particular drug can bind to two plasma protein each with one binding site. A list of definitions of symbols and abbreviations can also be found in reference 14. Before going forward, there are a few variables that need to be determined within the equation. The variables are *B_1_, B_2_, a_0_, a_1_, a_2_, q and r*.

$B_{1}= \frac{C_{bo_{1}}}{C_{mo_{1}}}= \frac{K_{1}C_{f}}{1+K_{1}C_{f}}$ (Equation 2)

$B_{2}=\frac{C_{bo_{2}}}{C_{mo_{2}}}= \frac{K_{2}C_{f}}{1+K_{2}C_{f}}$ (Equation 3)

$C_{to}= C_{f}+ C_{bo_{1}}+ C_{bo_{2}}$ (Equation 4)

$a_{o}= -\frac{C_{to}}{K_{1}K_{2}}$ (Equation 5)

$a_{1}= \frac{1+C_{mo1}K_{1}+C_{mo2}K_{2}-C_{to}(K_{1}+K_{2})}{K_{1}K_{2}}$ (Equation 6)

$a_{2}= C_{mo1}+C_{mo2}-C_{to}+\frac{1}{K_{1}}+\frac{1}{K_{2}}$ (Equation 7)

$q= \frac{3_{a_{1}}-a_{2}^{2}}{9}$ (Equation 8)

$r= \frac{9a_{2}a_{1}-27a_{0}-2a_{2}^{3}}{54}$ (Equation 9)

Using equations 2, 3, and 4 a system can be formed with three unknowns that can be solved for the free drug concentration (C_f_) in the sample. After determining the free concentration, the values of B_1_ and B_2_ can be calculated and plugged into equation 1 to compute the normalized concentration of a sample. Equations 5-9 are supplemental equations needed to determine all variables contained in the final normalizing equation. Equations 8 and 9 are derived as a function of the a_1_ coefficients and the numerical values of q and r can be used for deciding which equation to use for calculating the real root of the cubic equation.

If q^3^ + r^2^ ≥ 0 then the free concentration is given by:

$C_{f}= \sqrt[3]{r+ \sqrt{q^{3}+r^{2}}}+ \sqrt[3]{r-\sqrt{q^{3}+r^{2}}}- a_{2}/3$ (Equation 10)

If q^3^ + r^2^ < 0, then the free concentration is given by:

$C_{f}=2\sqrt{-q}\cos\left[ ArcCos\left( \frac{r}{\sqrt{-q^{3}}} \right)/3 \right]-\frac{a_{2}}{3}$ (Equation 11)

In the case of this research, q^3^ + r^2^ < 0 and the free concentration was determined by Equation 11.
